# Genetic and Epigenetic Markers of Lithium Response

**DOI:** 10.3390/ijms23031555

**Published:** 2022-01-29

**Authors:** Claudia Pisanu, Anna Meloni, Giovanni Severino, Alessio Squassina

**Affiliations:** 1 Section of Neuroscience and Clinical Pharmacology, Department of Biomedical Sciences, University of Cagliari, 09042 Cagliari, Italy; anna.meloni@unica.it (A.M.); severino@unica.it (G.S.); squassina@unica.it (A.S.); 2 Section of Functional Pharmacology and Neuroscience, Department of Surgical Sciences, Uppsala University, 75124 Uppsala, Sweden; 3 Department of Psychiatry, Faculty of Medicine, Dalhousie University, Halifax, NS B3H 2E2, Canada

**Keywords:** lithium, pharmacogenomics, pharmacogenetics, biomarker, personalized medicine, precision medicine, GWAS, methylation, epigenetic

## Abstract

The mood stabilizer lithium represents a cornerstone in the long term treatment of bipolar disorder (BD), although with substantial interindividual variability in clinical response. This variability appears to be modulated by genetics, which has been significantly investigated in the last two decades with some promising findings. In addition, recently, the interest in the role of epigenetics has grown significantly, since the exploration of these mechanisms might allow the elucidation of the gene–environment interactions and explanation of missing heritability. In this article, we provide an overview of the most relevant findings regarding the pharmacogenomics and pharmacoepigenomics of lithium response in BD. We describe the most replicated findings among candidate gene studies, results from genome-wide association studies (GWAS) as well as post-GWAS approaches supporting an association between high genetic load for schizophrenia, major depressive disorder or attention deficit/hyperactivity disorder and poor lithium response. Next, we describe results from studies investigating epigenetic mechanisms, such as changes in methylation or noncoding RNA levels, which play a relevant role as regulators of gene expression. Finally, we discuss challenges related to the search for the molecular determinants of lithium response and potential future research directions to pave the path towards a biomarker guided approach in lithium treatment.

## 1. Introduction

Bipolar disorder (BD) is a group of severe and disabling psychiatric disorders characterized by the recurrence of depressive and manic or hypomanic episodes, alternating with intervals of wellbeing [1]. The two main subtypes of BD include BD type I (BD I), defined by the presence of a manic episode, and BD type II (BD II), defined by the presence of a hypomanic episode and a major depressive episode [2].

With a prevalence of 1% in the population, early age at onset, high rate of medical comorbidities and increased risk of suicide, BD is associated with a substantial illness related disability and socioeconomic burden [3]. Pharmacological treatment is the mainstay in the acute phase of illness as well as in the prevention of recurrences. Specifically, the mood stabilizer lithium represents a cornerstone in the long term management of BD, due to its efficacy in the treatment of manic episodes, prevention of mood relapses and reduction of the risk of suicide [4,5]. However, only one third of patients show an excellent response to this drug, while the other two thirds show variable degrees of response, from partial to no response. Excellent responders to lithium have been suggested to represent a subphenotype and seem to share some clinical characteristics, such as a family history of BD and of lithium response, absence of rapid cycling and absence of psychotic symptoms [6,7]. However, low effect sizes and the limited predictive value of these characteristics, as well as the necessity of a long clinical observation to collect some of this information, do not allow a reliable and prompt identification of lithium responders in the clinical setting. Since lithium response has been suggested to be a heritable trait, a growing body of research has investigated the potential predictive value of genetic markers. In the last few years, studies have largely shifted from the assessment of candidate genes to the genome-wide approach, which is, however, still limited by several challenges related to the necessity of collecting large cohorts of patients with a deep clinical characterization. In this sense, international efforts, such as the International Consortium on Lithium Genetics (ConLiGen) [8], can provide a crucial contribution towards the identification of reliable markers of lithium response and the development of precision medicine approaches.

While genetic variants might explain part of the interindividual variability in lithium response, findings to date suggest that genetics can only explain a small proportion of this variability. To this regard, the exploration of epigenetic mechanisms appears fundamental to disentangling the missing heritability issue, as the study of these markers could help us significantly to elucidate how environmental variables can interact with genes and influence the probability of an individual to develop a disorder or show a clinical response to a drug. Moreover, other factors, often neglected in earlier studies but potentially contributing to the heterogeneity in lithium response, are now recognized as key variables to be accounted for in pharmacogenetic studies. Among others, gender could interact with genetic and epigenetic mechanisms underlying lithium response. Although the lifetime prevalence of BD is similar in both genders [9], women, compared to men, seem more likely to present the BD II subtype, rapid cycling, mixed episodes, as well as more frequent comorbidity with pain disorders, migraine and thyroid disorders [10,11,12,13,14,15]. In addition, menstrual cycle, pregnancy, the postpartum period and menopause may influence the course of the disease and the medication choice in women. A recent study suggested that men seem to be more likely to be prescribed lithium in Sweden, even after adjusting for bipolar subtype and the number of manic episodes [16]. While the majority of available studies do not suggest the existence of gender differences in lithium response [16], some studies suggested a worse [17] or a trend for a better response [18] in women. Gender might also affect the occurrence of side effects and reasons to interrupt lithium treatment [19]. For all these reasons, it is important to investigate whether gender might affect the interplay between lithium response and genetic or epigenetic factors.

In this narrative review, we describe studies investigating the genetic and epigenetic markers of response to the mood stabilizer lithium in peripheral cells or biofluids from patients with BD treated with lithium, or genetic/epigenetic markers affected by in vitro lithium treatment in human-derived cells. When available, we report whether discussed studies have assessed the potential impact of gender differences on reported findings.

## 2. Genetic Markers of Lithium Response

### 2.1. Candidate Gene Studies

Several studies have investigated the association between lithium response and different candidate genes. In this section, we discuss genes considered promising biomarkers based on the following arbitrary criteria: evidence provided by at least two studies, and inclusion of at least 50 patients (Table 1). Lithium acts on multiple signalling pathways, although it is not clear which of these effects underlie its clinical efficacy. Among these, lithium has been suggested to increase serotonin signalling, regulate intracellular calcium levels, as well as interfere with multiple second messenger systems, ultimately leading to the regulation of the transcription of different downstream targets (e.g., genes involved in neuroprotection, synaptic plasticity or regulation of circadian rhythms) [4]. Based on this evidence, many groups investigated the role of genes encoding proteins targeted by lithium, such as the glycogen synthase kinase 3 beta (GSK-3β) enzyme, as well as enzymes that play a role in inositol metabolism, such as inositol polyphosphate-1-phosphatase (INPP1) and inositol monophosphatase 2 (IMPA2). GSK-3β is a multifunctional kinase involved in different pathways, such as synaptic plasticity, apoptosis and circadian cycle [20,21], and is inhibited by lithium treatment both directly (via binding to the GSK-3 magnesium sensitive catalytic site) and indirectly (via increased serine phosphorylation of the enzyme) [22,23]. Different studies support the potential relevance of the *GSK-3* rs334558 variant. The CC genotype of this variant (false discovery rate [FDR] = 0.044 [24]) as well as the C allele (hazard ratio: 2.70, *p* = 0.007 [25]; *p* = 0.038 [26]), have been associated with better response to lithium treatment. One study found a trend for association between the CC genotype of rs6438552 and good response to lithium (odds ratio [OR] = 1.82, *p* = 0.08) [27], while another study reported that the TT genotype at rs6438552 was nominally associated with lower response (*p* = 0.044), although this association was not significant after multiple testing correction [24]. Gender was used as a covariate in the statistical analyses in most of these studies. A study investigating the association between this SNP and kidney function in patients with long term lithium treatment showed that the rs334558 TT genotype was significantly less frequent in men, while the TC genotype in women (*p* = 0.025) [28]. In this study, the rs334558 CC genotype was associated with higher urine specific gravity (*p* = 0.035), a parameter related to the concentration of excreted molecules in urine. Since lithium treatment can be associated with reduced urinary concentrating ability, the authors explored the hypothesis that this effect might be impacted by variants of the *GSK-3* gene. While no association between this SNP and other parameters of kidney function was reported, the small number of patients with the CC genotype (*n* = 7) might have affected this finding. Finally, the CT genotype at another variant (rs6438552) was associated with a significantly older age of onset than other genotypes in female patients (FDR = 0.042) [24].

Among genes implicated in the inositol metabolism, the rs2067421 variant, located in the *INPP1* gene, was associated with a better response to lithium in a study including 184 patients with BD (*p* = 0.04) [29]. In this study, no information regarding the number of women or whether gender was considered in the analyses was included. Support for a potential role of the same gene was provided by a more recent study from Mitjans and colleagues, showing a significant association between the rs3791809-rs4853694-rs909270 T-A-A haplotype and good lithium response (*p* = 0.018) [30]. The gene encoding the IMPA2 enzyme was also investigated. Specifically, the rs669838 C allele was associated with poor response (OR = 2.03, *p* = 0.021) in a study including 131 patients with BD in which analyses were adjusted for gender [30]. In a study including parent–offspring trios, Dimitrova and colleagues also showed that the rs3786282 C allele (*p* = 0.004) and the 599+97G/A A allele (*p* = 0.033) were overtransmitted among good responders [31].

Circadian rhythms have also been implicated among systems targeted by lithium. Promising results were provided for the nuclear receptor subfamily 1 group D member 1 gene (NR1D1), which encodes an enzyme that plays a role in the circadian regulation of microglial activation and neuroinflammation. The rs2314339 T allele was associated with a poor response (OR = 3.56, *p* = 0.0008) [32], while a nominal association between the rs2071427 A allele and a good response was reported (OR = 1.45, *p* = 0.05) [27].

Since disturbances in the neurotransmitter systems have been implicated in the pathogenesis of mood disorders, other studies focused on genes involved in neurotransmission and, specifically, in monoamines’ pathways. The s/s genotype of the serotonin transporter linked promoter region (5-HTTLPR), in the solute carrier family 6 member 4 (*SLC6A4*) gene, encoding the serotonin transporter, was associated with a worse response to lithium treatment in different independent studies (Table 1) [33,34,35]. On the other hand, another study reported an association between the s/s genotype and a better response to lithium (*p* = 0.04), but only among carriers of the C allele at the rs334558 variant in the *GSK-3* gene, suggesting a potential interaction between these two genes [36]. As regards potential gender differences, Serretti and colleagues reported an excess of 5-HTTLPR*l/s carriers among women (*p* = 0.01) [37]. In this study, the l/l genotype was associated with a higher incidence of nonresponders to lithium, exclusively among subjects who experienced ≤4 manic episodes before lithium (*p* = 0.005). The gene encoding the Catechol-O-Methyltransferase (COMT) enzyme, which is involved in the degradation of catecholamine neurotransmitters, was also investigated. The Val158Met Met allele was associated with a worse response to lithium treatment (*p* < 0.05, [33]) and mood stabilizers (*p* = 0.003, [38]), compared with the Val allele. Furthermore, the G allele and G/G genotype of the rs4532 variant of the dopamine D1 receptor (*DRD1*) gene, encoding the dopamine receptor D1, were associated with increased predisposition to BD as well as with poor response to lithium treatment [33,39].

Among the most investigated targets, the brain derived neurotrophic factor (*BDNF*) gene was implicated by different studies showing an association between the Val/Met genotype (*p* = 0.037, [40]) or the Met allele (lowest *p* = 0.002, [33,41]) of the Val66Met SNP (rs6265) and a good response. Interestingly, Wang and colleagues found that the Met allele had opposite effects on response to treatment with mood stabilizers based on BD subtype: the Val/Val carriers showed significantly lower response that the Val/Met carriers among patients with BD I (*p* = 0.025), while the opposite was observed in patients with BD II (*p* = 0.028) [42]. Another study found a significant interaction between microRNA 206 (*MIR206*) rs16882131 and *BDNF* Val66Met in the association with treatment response. In particular, individuals with *MIR206* TT + TC and *BDNF* Met/Met genotypes had a significantly lower mean treatment score than those with *MIR206* CC and *BDNF* Met/Met + Val/Met genotypes (*p* = 0.018), as well as those with *MIR206* CC and *BDNF* Val/Val genotypes (*p* = 0.013) [43]. As regards to other BDNF variants, Dmitrzak-Weglarz and colleagues reported the rs988748 G allele to be more frequent among lithium poor responders, compared with nonresponders (*p* = 0.047) [41]. Another group observed a trend for association between the CT genotype (*p* = 0.057) and T allele (*p* = 0.065) of the -270C/T polymorphism and positive response to lithium treatment [40]. Neurotrophic receptor tyrosine kinase 1 (*NTRK1*) gene was also investigated. The rs1387923 SNP was associated with lithium response after adjusting for history of suicidal ideation (adjusted *p* = 0.027) [29], while rs2769605 with response to mood stabilizers (*p* = 0.01) [44].

Among other genes, the -116CC or -116CG genotypes of the *XBP1* gene, encoding the X-box binding protein 1, were associated with a better response to lithium treatment compared with the -116GG genotype (*p* = 0.049, [45]; OR = 3.00, *p* = 0.037 [46]). Finally, two studies provided significant results for the calcium voltage-gated channel auxiliary subunit gamma 2 (*CACNG2*) gene (Stargazin). Specifically, the rs2284017 (cohort 1, OR = 0.35, *p* = 0.029; cohort 2, OR = 0.38, *p* = 0.045 [47]) and rs140040 (OR = 1.73, *p* = 0.003 [48]) SNPs were associated with lithium response.

Taken together, these studies provided promising results supporting the potential association between some genetic variants and lithium response. However, as will be discussed in the next section, scarce support for these candidate genes has been provided by GWAS, to date. Among the potential reasons underlying these discrepancies there is the scarce knowledge on lithium’s mechanism of action, which makes the identification of genes underlying its clinical efficacy challenging, as well as the large heterogeneity of these studies in terms of design, disease subtypes as well as the characterization of lithium response. While the large majority of available studies did not observe significant gender differences, not all studies reported the number of men and women included, or specified whether gender was included in the analyses. Importantly, for the large majority of these genes, negative findings as regards their association with lithium response have also been reported [29,42,46,49,50,51,52].

### 2.2. Genome-Wide Association Studies

In the last ten years, research on genetic determinants of lithium response moved from candidate genes to the genome-wide approach. To date, five GWAS on lithium response have been conducted (Table 2). Among these, three evaluated response to lithium using the validated Retrospective Criteria of Long-Term Treatment Response in Research Subjects with Bipolar Disorder (Alda scale) [7], while the other two used different criteria (Table 2). Briefly, the Alda scales measures the change in illness episodes during lithium therapy using two criteria: criterion A quantifies the clinical improvement with a score from 0 (no change in disease severity) to 10 (complete remission), while criterion B evalautes potential confounding factors. The total score is computed by subtracting the B score from the A score. Based on a previous inter-rater reliability study [54], two main phenotypes for lithium response have been proposed: a dichotomous (subjects with a total score of 7 or higher are considered to be responders) and a continuous phenotype (based on the A score, excluding all individuals with a B score greater than 4). Two studies reported genome-wide significant results. The largest of these studies was conducted by ConLiGen and included data for 2563 patients with BD collected from 22 ConLiGen sites [55]. The study highlighted a region on chromosome 21 containing four SNPs in linkage disequilibrium (LD) associated with lithium response evaluated as a continuous trait (lowest *p* = 3.3 × 10^−9^, rs74795342). Importantly, these results were supported by findings obtained in an independent prospective cohort of 73 patients in which heterozygote carriers of the alleles associated with poorer lithium response in the discovery cohort showed a higher rate of relapses compared with carriers of the alternative alleles (hazard ratio = 3.8, *p* = 0.033) [55]. The other GWAS able to report genome-wide significant results was conducted by Chen and colleagues and included data for 294 BD patients of Han Chinese descent and two replication cohorts including 100 and 24 patients, respectively [56]. Lithium response was evaluated with the Alda scale using four cutoff points of the total score to identify responders (4 to 5 points, 5 to 6 points, 6 to 7 points and 7 to 8 points). Two SNPs in LD located in the intronic region of the glutamate decarboxylase like 1 (*GADL1*) gene (rs17026688, *p* = 5.50 × 10^−37^; rs17026651, *p* = 2.52 × 10^−37^) were associated with lithium response in the discovery sample (strongest association was found using the cutoff point of 5 to 6) as well as in the two replication samples. However, subsequent independent studies were not able to replicate these findings [57].

### 2.3. Post-GWAS Approaches

While GWAS have uncovered throusands of genetics variants associated with different complex traits, including susceptibility for psychiatric disorders [61,62,63], the identification of causal genetic variants is challenging. Cross-trait analyses, gene based analyses, the integration of different types of omic data and transcription-wide association studies are only some of the computational approaches that can be used to build on GWAS knowledge, to gain insights into the molecular mechanisms underlying the observed associations. In the case of lithium response, this research is still in its infancy. However, some of these approaches, such as cross-trait analyses and polygenic risk score (PRS), have started to be applied to this trait [64,65]. Recent studies that leveraged the GWAS dataset from the ConLiGen consortium as well as larger datasets on severe psychiatric disorders from the Psychiatric Genomics Consortium, suggested a high genetic load for schizophrenia (OR for lithium response ranging from 3.46 at the first decile to 2.03 at the ninth decile, compared with the patients in the 10th decile of schizophrenia risk) [66], major depressive disorder (lowest vs highest PRS quartiles OR = 1.54) [67] or attention deficit hyperactivity disorder (β = -0.14; *p* = 0.01) [68] to be associated with poor lithium response. In addition, a PRS combining variants associated with either schizophrenia or depression was recently shown to be able to improve the prediction of lithium response compared to single disorder PRS (proportion of phenotype variance explained by combined PRS: partial R^2^ = 0.91%; schizophrenia–PRS: partial R^2^ = 0.82%; depression–PRS partial R^2^ = 0.47%) [69]. In the ConLiGen cohort, patients in the highest decile for the combined PRS had 2.5 times higher odds of being poor responders compared with patients in the lowest decile [69]. These approaches are based on pleiotropy, i.e., a condition in which the same gene affects multiple phenotypes simultaneously. It is important to mention that GWAS, to date, have mostly included participants of European ancestry. Due to this, the predictive accuracy of the PRS constructed using these data is lower in individuals from other populations [70]. In order to address this problem, there is now growing effort to conduct GWAS including participants from worldwide populations.

Building on the observation that the most significant finding of the cross trait GWAS between lithium response and schizophrenia was related to the human leukocyte antigen (HLA) cluster, which is hosted by the major histocompatibility complex (MHC) region, a recent study conducted a deeper investigation by imputing the HLA class-I and class-II genetic variants corresponding to HLA amino acids and classical alleles in a subset of the ConLiGen dataset [71]. While no significant results were obtained after multiple testing correction, the best signal was observed for amino acid variants belonging to the HLA-DRB1*11:01 classical allele (FDR < 0.09). Alanine or leucine, at position 74 of the HLA-DRB1 heavy chain, were associated with a good response (p = 1.9 × 10^−3^, FDR = 0.27), while arginine or glutamic acid (p = 3.5 × 10^−3^, FDR = 0.27) with a poor response. Being these variants associated with inflammatory/autoimmune processes, results from this study support the hypothesis that the HLA mediated inflammatory status may play a role in lithium response. Finally, based on growing evidence supporting the potential role of epigenetic determinants in the mechanism of action of mood stabilizers [72], another study conducted an in depth analysis of variants located in microRNAs (miRNAs). Specifically, Reinbold and colleagues tested the association between lithium response and variants located in nine candidate miRNAs in the ConLiGen dataset [73]. In addition, the authors conducted a gene based analysis for all known miRNA coding genes. While no statistically significant results were reported after multiple testing correction, the study highlighted 15 miRNAs nominally associated with both definitions of lithium response, with miR-633 (*p* = 9.8 × 10^−4^) and miR-607 (*p* = 5.8 × 10^−4^) showing the strongest association with the continuous and the dichotomous phenotype, respectively.

## 3. Epigenetic Markers of Lithium Response

Epigenetic mechanisms might mediate the interaction between susceptibility genes and environmental factors [74]. These mechanisms regulate gene expression through heritable modifications that do not affect DNA sequence, such as methylation or hydroxymethylation at the 5-position of cytosine residues of CpG dinucleotides, modifications of the histone proteins as well as the regulation of gene expression via noncoding RNAs [75]. DNA methylation consists of the addition of methyl groups to a cytosine-guanine dinucleotide (CpG) and is catalysed by the DNA methyltransferases enzymes. In the majority of cases, DNA methylation at gene promoters is associated with the transcriptional silencing. However, CpG dinucleotides can also interfere with splicing or induce increased gene expression (for instance, via blocking the binding of repressive proteins) [76]. Noncoding RNAs comprise products from the transcribed portion of the genome that are not translated into proteins. Among the different noncoding RNAs, small noncoding RNAs (miRNA) are small (~18–25 nucleotides) single stranded RNA molecules involved in the post-transcriptional regulation of gene expression via binding to untranslated regions of mRNAs. Genes targeted by miRNAs play a crucial role in processes such as neural development, neuronal function and synaptic plasticity. In addition, miRNAs can also affect other epigenetic mechanisms by targeting enzymes responsible for epigenetic modifications, such as DNA methyltransferases or histone deacetylases [77]. Long noncoding RNAs (lnc-RNA) are RNA molecules longer than 200 nucleotides. While much more prevalent compared to mRNAs, the exact function of the majority of lnc-RNAs has not been characterized yet. However, a number of lnc-RNAs has been suggested to be involved in key cellular functions such as neurogenesis or neuroplasticity [78]. In the following sections, we will focus on the epigenetic mechanisms that were investigated in studies on lithium exposure or clinical response conducted in human-derived cell lines, i.e., DNA methylation and changes in the levels of noncoding RNAs.

### 3.1. DNA Methylation and Lithium Exposure or Clinical Response

#### 3.1.1. Candidate Gene Studies

An overview of studies investigating changes in DNA methylation levels based on lithium response or exposure to lithium treatment is shown in Table 3. Considering the limited number of avialable studies, no arbitrary criteria were applied to restrict the number of studies to discuss. Candidate gene studies mostly focused on the *BDNF* gene, based on different lines of evidence supporting its potential role in the pathogenesis of severe mental disorders [79,80,81]. D’Addario and colleagues measured the degree of DNA methylation at the *BDNF* promoter region, as well as mRNA levels of the gene, in peripheral blood mononuclear cells (PBMC) from 94 patients with BD and 52 controls matched for age, gender and origin. The authors observed that patients with BD II but not BD I showed a significant hypermethylation of the *BDNF* promoter region and, consistently, reduced *BDNF* gene expression compared with controls (*p* < 0.05 for both) [82]. In addition, patients under treatment with lithium and valproate showed a significant hypomethilation compared with patients treated with other drugs (*p* < 0.05). A similar though nonsignificant trend for the hypomethylation of the *BDNF* promoter in patients treated with lithium and valproate compared with other drugs was observed in a subsequent study by the same group, including 61 patients with BD type I, 50 with BD II and 43 with major depressive disorder on stable pharmacological treatment [83]. In this study, age and gender did not seem to exert a significant effect on methylation levels of the *BDNF* promoter. In a subsequent study including 54 patients with BD I, 45 with BD II and 42 controls matched for gender, age and origin, the authors reported an increase in DNA methylation at the promoter of the prodynorphin (*PDYN*) gene, which encodes a precursor of the dynorphin peptide [84]. Besides reporting higher *PDYN* methylation in patients with BD II compared with controls (*p* < 0.05), the authors observed lower methylation levels in patients treated with lithium and anticonvulsants compared with other drugs (*p* = 0.015), as well as a positive correlation in promoter DNA methylation between *PDYN* and *BDNF* (*p* = 0.007) [84].

Another candidate gene study, including 151 patients with BD and 66 controls, used pyrosequencing to assess the methylation status of two CpG islands located in the first exon and the 5′ region of the aryl hydrocarbon receptor nuclear translocator like (*ARNTL*) clock gene (PS2 and cg05733463, respectively) [85]. This gene was selected based on a large body of research supporting the role of circadian rhythm dysfunctions in the pathogenesis of BD [86]. Analyses were adjusted for different relevant covariates, including age, gender, BMI, smoking, lithium (26.4%) and anticonvulsants (28.9%) intake. The authors observed higher methylation levels at cg05733463 (*p* < 0.001) and lower at POS1 (*p* = 0.018) of the CpG island PS2 in patients with BD compared with controls [85]. In addition, a significant association between methylation of cg05733463 in the *ARNTL* gene and lithium (*p* = 0.005) or anticonvulsant (*p* = 0.002) intake was observed, although the direction of effect was not reported.

#### 3.1.2. Studies on Global or Genome-Wide Methylation Levels

A genome-wide study conducted in human neuroblastoma SK-N-SH cells suggested that lithium might exert extensive effects on methylation [92]. The authors examined the effects of in vitro treatment for 8 days with different mood stabilizers (lithium, 0.6 and 1.2 mM; valproate 0.3 and 0.6 mM; carbamazepine, 0.05 and 0.1 mM), showing a larger number of genes to be differentially methylated in cells treated with different doses of lithium (345 genes were hypermethylated and 138 hypomethylated) compared to valproate and carbamazepine (64 and 64 genes were hypermethylated, 36 and 14 genes were hypomethylated, respectively). Genes commonly altered by different mood stabilizers were enriched for gene ontology (GO) terms related to developmental processes, while genes specifically altered by lithium were enriched for terms related to neuronal functions, such as cell–cell signalling, synaptic transmission and neurotransmitter receptor activity [92].

Few studies investigated the association between exposure to lithium and global methylation levels in cells derived from patients with BD using different methods, such as luminometric methylation assay (LUMA) [87] or ELISA [88,90]. While the LUMA method is based on restriction enzymes and pyrosequencing reactions, ELISA based methods detect methylated cytosines through incubation steps with monoclonal antibodies and quantify them through a colorimetric/fluorometric reaction. Burghardt and colleagues showed lower global methylation levels in leukocytes from 84 patients with BD treated with second generation antipsychotics, compared to 31 treated with mood stabilizers, 35% of which were treated with lithium (*p* = 0.04) [87]. Analyses were adjusted for smoking status, age, gender, race, folate level and waist to hip ratio. In line with previous studies, the authors reported that smoking status and insulin resistance influenced global methylation levels. Gender did not provide a significant contribution to the model. Huzayyin and colleagues evaluated the effect of in vitro lithium treatment on global methylation levels as well as markers of oxidative stress in lymphoblastoid cell lines (LCL) from patients with BD with excellent response to lithium treatment, their affected and unaffected relatives and controls [90]. While the number of male and female patients was reported, analyses do not seem to have been adjusted for gender. At baseline, global methylation levels were lower in patients with BD (*p* = 0.021) and their affected (*p* = 0.006) or unaffected (*p* = 0.003) relatives compared with controls. After in vitro treatment with lithium 0.75 mM for seven days, methylation levels increased in relatives but not in patients with BD or controls. Finally, Backlund and colleagues measured global methylation levels in leukocytes from 29 patients with BD treated with lithium monotherapy, 32 with lithium and either valproate (*n* = 11) or antipsychotics (*n* = 22) and 26 controls. Analyses were adjusted for age and gender. In addition, only patients with a nonsmoker status during the year before enrollment were included. Patients under lithium monotherapy showed decreased global DNA methylation compared to both patients treated with lithium + valproate or controls (*p* = 0.036). Gender was significantly associated with methylation levels in the analyses comparing patients on lithium monotherapy with patients on lithium + valproate, but not in the comparison between patients on lithium monotherapy with controls. On the the other hand, no association between methylation levels and response to lithium assessed using the Alda scale was observed [88]. Another study from Higgins and colleagues used a complementary approach to provide evidence supporting a role of epigenetic modifications in the mechanism of action of lithium [93]. This study reported that genetic variants previously associated with lithium response in GWAS showed concordance with regulatory elements annotated by the Epigenome roadmap consortium, suggesting that genetic determinants of lithium response might interfere with regulatory and epigenetic mechanisms.

To our knowledge, only two studies evaluated genome-wide metylation profiles in patients with BD characterized for exposure [89] or response to lithium [91]. The first study examined whole blood DNA methylation signatures of exposure to different psyhcotropic drugs in a sample including 172 patients with BD, 55% of which were women, treated with the mood stabilizers lithium, valproate, carbamazepine or lamotrigine, or with the antipsychotics olanzapine and quetiapine [89]. All analyses to evaluate the association between medication types and methylation levels were adjusted for age, gender, smoking status and cell type composition. The authors represented DNA methylation levels using principal components (PC) and weighted gene coexpression network analysis (WGCNA) modules. Valproate was significantly associated with higher values of the second PC of methylation levels (*p* = 0.02), while olanzapine and lithium with lower values (*p* = 0.05 for both) on this PC. Lithium was not significantly associated with any WGCNA module. Overall, the study suggested that treatment with valproate and quetiapine, but not lithium, to be significantly associated with altered methylation signatures after adjustment for drug related changes on cell type composition.

In the most recent study, Marie-Claire and colleagues (2020) analyzed whether prophylactic response to lithium assesssed retrospectively is associated with specific DNA methylation profiles [91]. In this study, the authors measured genome-wide methylation using mehylCap-seq in peripheral blood cells from 26 euthymic patients with BD type 1 (14 men and 12 women), of whom 15 were excellent responders to lithium and 11 nonresponders. Response to lithium was retrospectively assessed using the Alda Scale [7]. The two groups were matched on potential confounding factors known to affect methylation levels such as age, gender, BMI and smoking status, although no significant effect for these factors was reported [91]. The authors identified 111 differentially methylated regions (DMR) associated with lithium response (FDR < 0.05), among which 66 were found not to be affected by current lithium treatment or comedication status and used for further analyses. The authors found that a signature including seven DMRs, selected using sparse partial least square discriminant analysis, was able to discriminate between responders and nonresponders with a sensitivity of 0.818 and a specificty of 0.867. Overall, findings supporting the potential role of changes in DNA methylation in lithium’s mechanism of action and clinical response are promising. While the majority of studies adjusted analyses for some of the factors known to be able to affect methylation levels, including gender, the limited number of studies, as well as their small sample size, calls for further research and replication in independent cohorts.

### 3.2. Noncoding RNAs and Lithium Exposure or Response

A limited number of studies evalauted noncoding RNA levels in cells derived from patients with BD treated with lithium and only three of them characterized patients for lithium response (Table 4). Chen and colleagues evaluated the levels of 13 miRNAs in LCLs derived from 10 BD I and 10 discordant siblings matched by gender. Therein, miRNA levels were measured before and after in vitro treatment with lithium 1 mM for 4, 8 and 16 days [94]. In this study, no significant effect of gender was detected. The four miRNAs miR-221, miR-152, miR-155 and miR-34a were upregulated by lithium both at day 4 and 16 (*p* < 0.05). Interestingly, two of these miRNAs (miR-221 and miR-34a) were previously found to be affected by lithium treatment in rat’s hippcampus, though with the opposite direction of effect [95]. A subsequent study with a candidate miRNA design evaluated plasma levels of miR-134 in 21 patients with BD during a manic episode (14 men and 7 women), before and after 2 and 4 weeks of treatment with different mood stabilizers and antipsychotics, as well as controls matched by age and gender [96]. This miRNA was chosen based on its potential involvement in the regulation of dendritic spine volume and synapse formation. Plasma miR-134 levels were lower in patients compared with controls at baseline and significantly increased after 4 weeks of treatment (*p* < 0.001 for both) [96]. In addition, levels of this miRNA were negatively correlated with the severity of manic symptoms measured with the Beck–Rafaelsen Manic Scale (BRMS) (*p* = 0.009). While 14 patients were treated with lithium, since no stratified analyses were conducted, it is not possibile to elucidate whether this effect might be specifically associated with lithium treatment. Another study observed a nonsignificant trend for higher expression of let7-c in LCLs from ten patients with BD nonresponders compared with ten responders to lithium (fold change [FC]: 1.6, *p* = 0.1) [97]. No significant effect of in vitro treatment with lithium 1–15 mM on this miRNA was reported in any group [97]. On the other hand, another study reported several members of the let-7 family to be downregulated by in vitro treatment with lithium 1 mM for 1 week in LCLs derived from both responders and nonresponders [98]. Finally, two studies measured genome-wide miRNA levels in LCLs from patients with BD characterized for lithium response [99] and suicide behavior [100], respectively. The first study used next generation sequencing and identified two clusters of miRNAs with a significant differential expression between responders and non-responders. Among these, miR-320a (FC = 0.55, FDR = 3.8 × 10^−8^) and miR-155 (FC = 2.27, FDR = 0.003), which were found to be down- and upregulated in responders, respectively, were validated with quantitative reverse transcription PCR (qRT-PCR) [99]. The second study compared LCL genome-wide miRNA levels between 7 patients with BD who died by suicide (3 women) and 11 patients with low risk of suicide (7 women) [100]. The two most significant miRNAs were validated with qRT-PCR in the same sample and in 12 controls. The study found miR-4286 to be increased in baseline (FC = 1.16; FDR = 0.0018) or lithium treated (FC = 1.17; FDR = 0.0013) LCLs and miR-186-5p to be decreased in lithium treated LCLs (FC = 0.96, FDR = 0.0045) from patients who died by suicide compared with patients at low risk and controls [100]. While these miRNAs were not affected by in vitro treatment in LCLs, treatment with lithium significantly reduced miR-4286 expression in human neural progenitor cells (FC = 0.31, *p* = 0.038) [100]. With few exceptions, although in most studies participants were matched by gender, the large majority of studies did not evaluate the effect of gender in the association between miRNA levels and lithium response.

To conclude this section, we discuss studies that evaluated the potential role of miRNAs in other human-derived cells. Croce and collegues compared the effects of exposure to lithium or valproate on the expression of miR-30a-5p, a miRNA involved in regulation of the neuroprotective factor BDNF, in an in vitro model of neurodegeneration (a human neuroblastoma cell line exposed to neurotoxic concentration of L-glutamate 2 mM) [101]. While pretreatment with both mood stabilizers abolished the reduced cell survival observed after treatment with L-glutamate, possibly via an increase of BDNF protein levels, this effect did not seem to be mediated by changes in miR-30a-5p expression. In fact, at 24 h of incubation, both glutamate (*p* < 0.01) as well as the two mood stabilizers + glutamate (*p* < 0.05) induced an increase in miR-30a-5p expression, while a nonsignificant increase was observed for pretreatment with lithium + valproate. At 48 h, both glutamate (*p* < 0.01) or lithium + valproate (*p* < 0.001) induced a decrease in miR-30-5p levels compared with untreated cells [101]. Therefore, changes in miR-30a-5p expression induced by lithium do not seem to be related to neuroprotective effects. Finally, a more recent study evaluated with whole transcriptome sequencing the effects of in vitro treatment with lithium 1 mM for 1 week on genome-wide RNA levels in neuroblastoma neuronal cultures [102]. The authors analyzed results by RNA length and type, showing that shorter RNA sequences were predominant among RNAs affected by lithium. Specifically, lithium was more likely to influence noncoding small nucleolar RNAs (snoRNAs), compared to miRNAs (*p* < 0.05) [102].

## 4. Discussion

The effort to identify genetic and epigenetic determinants of response to lithium in patients with BD has been remarkable in the last two decades. Nevertheless, despite the fact that the new technological strategies and bioinformatic approaches allowed a great step forward compared to the early stages of pharmacogenetic research, overall only few targets have been replicated across studies. Even when findings were replicated by two or more independent candidate gene studies, these results found scarce support in genome-wide scans. This scenario has been determined by different factors, but it has become clear that the exploration of such a complex and heterogeneous trait such as response to lithium requires integrated approaches to be successful.

Post-GWAS methods highlighted the potential of cross-trait analyses, allowing the leveraging of larger datasets on well studied phenotypes genetically correlated with lithium response, such as schizophrenia and major depressive disorder. However, the search for biological markers of response to lithium poses relevant challenges, some shared with other complex phenotypes, some specific for this trait. As in the cases of other complex phenotypes, heterogeneity among individuals due to, e.g., ancestry, diagnosis subtypes, severity of illness and medical comorbidities, might act as a confounding factor, requiring great effort, deep phenotyping and large samples to be overcome or at least accounted for in the analytical models. This is further tangled by the peculiarities of lithium pharmacokinetics and pharmacodynamics. Indeed, as an ion, lithium is not metabolized by liver enzymes, which typically represent the most widely and likely informative targets currently available in the pharmacogenetics of psychotropic medications. Moreover, the wide range of molecular pathways affected by lithium, as well as our still scarce knowledge of the molecular mechanisms underlying its therapeutic effects, limits our ability to focus on specific candidate targets. Besides, studies on the prophylactic effect of long term lithium treatment have been also hampered by the long time of observation required to clinically assess response, since this represents an obstacle to the collection of large samples of well characterized patients, which are usually required for genome-wide studies.

The paucity of replicated findings, and the limited variability explained by genetic variations alone, highlighted the importance of extending our investigation to molecular targets other than DNA variants, such as epigenetic mechanisms or the microbiome. It also supports the need to account for the contribution of clinical and sociodemographic features that have often been neglected in earlier studies, as is the case for gender.

Of the studies including gender based analysis in lithium response, the majority did not report significant gender differences. However, a high number of studies did not specify whether gender was included in the analyses. Since it is known that gender may influence DNA methylation levels [103], most of the studies investigating this trait included gender among adjusting factors, together with age and smoking. As for lithium response, only one study showed that gender was significantly associated with methylation levels in patients on lithium monotherapy compared with patients on lithium + valproate [88], while the few other studies reported no significant association between gender and either global methylation [87] or genome-wide methylation levels [91]. Overall, since a high number of studies did not include gender in the analytical models and did not conduct stratified analyses based on gender, a significant contribution of this factor to the relationship between genetic or epigenetic determinants and lithium response cannot be excluded.

As mentioned earlier, it is now clear that genetic variants can only explain a small proportion of the variability in lithium response, and that other mechanisms are involved. Among these, epigenetics appears to be a promising candidate that can significantly contribute to shedding light on the complex molecular mechanisms involved in lithium efficacy. Indeed, it can be speculated that studies integrating multiple omic data, such as genome-wide genotyping, gene/protein expression and methylation data, might allow us to gain a clearer understanding of the still elusive relationships between molecular determinants of lithium response. However, very few studies, generally including small numbers of patients, have started to apply this approach. Nevertheless, recent efforts leveraging large and well powered studies from genetically correlated traits have already started to generate novel insights on molecular mechanisms putatively underlying lithium’s clinical efficacy. On the other hand, the matter of discerning between unspecific and clinically relevant molecular mechanisms of lithium remains too complex to be addressed through clinical studies. To this regard, a significant contribution has been provided by in vitro studies taking advantage of neurons derived from induced pluripotent stem cells (iPSC) from patients. Findings from these studies expand our knowledge on the mechanism of action of lithium, also supporting hypotheses that were generated through different strategies by earlier studies. This is for instance the case of mitochondria [104]. Indeed, a recent study explored the effect of in vitro treatment with lithium on oxygen consumption rate (OCR) and glycolytic rate (two indexes of mitochondrial respiratory function) in iPSC derived neural precursor cells (NPCs) [105]. Cells were derived from three patients responders to lithium, three patients not exposed to lithium and three controls. Intriguingly, while the authors found a nonsignificant tendency for lower OCR in cells derived from lithium responder patients compared with controls, in vitro treatment with lithium improved OCR levels by enhancing maximal respiration and reserve capacity exclusively in cells derived from lithium responders [105].

Another promising area of investigation is represented by the noncoding portion of our genome. Based on the growing number of studies supporting the hypothesis that ncRNA levels might play a role in the efficacy of lithium, relevant results might be provided by studies investigating miRNA levels in peripherally isolated exosomes. These extracellular vesicles are secreted by several types of cells (including neurons, glial cells and neural precursors) into the extracellular matrix and play a crucial role in intercellular communication and signal transmission. Neural exosomes can cross the blood–brain barrier, thus potentially allowing to identify brain relevant biosignatures of disease and drug response in a noninvasive way. While a recent study provided interesting preliminary evidence on the potential use of miRNAs from extracellular vesicles as biomarkers of BD [106], the potential contribution of exosomes and other extracellular vesicles as a source of biomarkers for lithium response has not been investigated yet.

To conclude, the molecular mechanisms underlying lithium response are still elusive, and findings are still far from being clinically useful to guide lithium therapy. A better understanding of its mechanism of action and the identification of the demographic and clinical features that might allow to identify more homogenous subgroups of patients are the key to a better management of lithium treatment in BD. Findings from recent efforts appear promising, and suggest that the leveraging of pleiotropy, integration of clinical data with different omic traits as well as novel sources of biomarkers such as exosomes, might allow to shed further light on the genetic and epigenetic determinants of response to long term lithium treatment, leading us closer to precision medicine in BD.

## Figures and Tables

**Table 1:** Candidate genes for which association with lithium response has been provided by at least two studies including at least 50 patients with BD.

Gene	Sample	Results	Ref.
*GSK-3β*	131 BD patients (62 F)	Association between rs1732170-rs11921360-rs334558 C-C-A haplotype and good response (*p* = 0.001)	[30]
	138 BD patients (67 F)	Association between rs334558 TT genotype and poor response (FDR = 0.044)	[24]
	88 BD patients (61 F)	Association between rs334558 C allele and good response (*p* = 0.038)	[26]
	81 patients with bipolar or unipolar depression (46 F)	Association between rs334558 C allele and better response to lithium augmentation (hazard ratio: 2.70, *p* = 0.007) compared with the TT genotype. No significant gender differences in the frequency of genotypes	[25]
	78 BD patients (53 F)	Association between rs334558 CC genotype and higher urine specific gravity (*p* = 0.035). The TT genotype was significantly less frequent in men, while the TC genotype in women (*p* = 0.025)	[28]
	138 BD patients (67 F)	Nominal association between rs6438552 TT genotype and poor response (*p* = 0.044). CT genotype was associated with a significantly older age of onset in female patients (FDR = 0.042)	[24]
	282 BD patients	Trend for association between SNP rs6438552 and good response (OR = 1.82, *p* = 0.08). All analyses were adjusted for gender	[27]
*INPP1*	184 BD patients	Association between rs2067421 and good response after accounting for euphoric/dysphoric mania and history of suicidal ideation (*p* = 0.04)	[29]
	131 BD patients (62 F)	Association between the rs3791809-rs4853694-rs909270 T-A-A haplotype and good response (*p* = 0.018)	[30]
*IMPA2*	116 BD parents-offspring trios	Overtransmission of the rs3786282 C allele (*p* = 0.004) and the 599+97G/A A allele (*p* = 0.033) among good responders	[31]
	131 BD patients (62 F)	Significant association for rs669838 C allele and poor response (OR = 2.03, *p* = 0.021)	[30]
*BDNF*	88 BD patients (53 F)	Association between the Val66Met Val/Met genotype and excellent response (*p* = 0.037). Higher proportion of male patients among non responders (62.5%) compared with other response groups	[40]
	108 BD patients (66 F)	Association between the Val66Met Met allele and excellent response (*p* = 0.002)	[41]
	101 BD patients (58 F)	Lower prevalence of Val66Met Val/Val genotype and Val allele in responders (*p* < 0.05)	[33]
	342 BD patients (190 F)	Met allele had opposite effect on treatment response based on BD subtype: the Val/Val genotype was associated with poor or good response in patients with BD I (*p* = 0.025) or BD II (*p* = 0.028), respectively	[42]
	280 BD patients (156 F)	Interaction between *MIR206* rs16882131 and *BDNF* Val66Met: carriers of *MIR206* T/T+TC and *BDNF* Met/Met genotypes had a significantly lower treatment score than those with *MIR206* CC and *BDNF* Met/Met+Val/Met genotypes (*p* = 0.018) as well as those with *MIR206* CC and *BDNF* Val/Val genotypes (*p* = 0.013)	[43]
	108 BD patients (66 F)	rs988748 G allele was more frequent among poor responders compared with non responders (*p* = 0.047)	[41]
	88 BD patients (53 F)	Trend for association between the 270C/T CT genotype (*p* = 0.057) and T allele (*p* = 0.065) and excellent responders compared with non-responders	[40]
*NTRK1*	184 BD patients	rs1387923 (adjusted *p* = 0.027) was associated with lithium response, accounting for history of suicidal ideation.	[29]
	284 BD patients (157 F)	Significant allelic association between rs2769605 and treatment response to mood stabilizers in patients with BD I (*p* = 0.01)	[44]
*SLC6A4*	201 BD patients (118 F)	Association between the 5-HTTLPR s/s genotype and worse response (*p* = 0.009). No significant gender differences	[35]
	122 BD patients (90 F)	Association between 5-HTTLPR s/s genotype and good lithium response, only in carriers of the C allele at the rs334558 variant in the *GSK-3* gene (*p* = 0.04)	[36]
	111 BD patients (68 F)	Interaction between 5-HTTLPR and *BDNF* Val66Met variants: 5-HTTLPR s carriers having the Val/Val genotype were significantly more frequent in non responders compared with excellent or/and poor responders (*p* = 0.004)	[34]
	101 BD patients (58 F)	Association of the 5-HTTLPR s allele with poor response (*p* < 0.05)	[33]
	122 BD patients (57 F)	Association of haplotype consisting of the s allele of 5-HTTLPR and 10 repeat allele of STin2 with lithium response (*p* = 0.01). Analyses were adjusted for gender	[53]
	83 BD patients (55 F)	Statistically significant excess of the l/s genotypes among females. The l/l genotype was associated with a higher incidence of nonresponders to lithium, exclusively among subjects who experienced ≤ 4 manic episodes before lithium (*p* = 0.005)	[37]
*COMT*	144 BD patients (92 F)	Association between the Val158Met Val allele and good response to mood stabilizers (*p* = 0.003)	[38]
	101 BD patients (58 F)	Association between the Val158Met Met allele and poor response (*p* < 0.05)	[33]
*DRD1*	92 BD patients (53 F)	Association between the rs4532 G allele and the GG genotype with poor response (*p* < 0.05)	[39]
	101 BD patients (58 F)	Association between the rs4532 GG genotype and poor response (*p* < 0.05)	[33]
*NR1D1*	282 BD patients	Nominal association between the rs2071427 A allele and good response (OR = 1.45, *p* = 0.05). Analyses were adjusted for gender	[27]
	170 BD patients	Association between the rs2314339 T allele and poor response (OR = 3.56, *p* = 0.0008). Analyses were adjusted for gender	[32]
*CACNG2*	Retrospective: 286 BD patients (138 F); Prospective: 68 BD patients (8 F)	Nominal association between rs140040 (OR = 1.73, *p* = 0.003) and good response. Analyses were adjusted for gender	[48]
	383 BD patients	Significant associations between the rs2284017 SNP and lithium response in both cohort 1 (OR = 0.35, *p* = 0.029) and cohort 2 (OR = 0.38, *p* = 0.045). No gender differences in lithium response	[47]
*XBP1*	56 BD patients (28 F)	Patients with the -116GG genotype showed a significantly smaller proportion of responders, compared to -116CC or -116CG patients (*p* = 0.049)	[45]
	66 BD patients (38 F)	Lithium treatment is more effective among carriers of the -116C allele compared with the G allele (OR = 3.00, *p* = 0.037)	[46]

BD I, bipolar disorder type 1; BDNF, brain-derived neurotrophic factor; CACNG2, calcium voltage-gated channel auxiliary subunit gamma 2; COMT, catechol-O-methyltransferase; DRD1, dopamine D1 receptor; FDR, false discovery rate; GSK-3β, glycogen synthase kinase 3 beta; HC, healthy controls; F, female; IMPA2, inositol monophosphatase 2; INPP1, inositol polyphosphate-1-phosphatase; NR1D1, nuclear receptor subfamily 1 group D member 1; NTRK1, neurotrophic receptor tyrosine kinase 1; OR, odds ratio; SLC6A4, solute carrier family 6 member 4; SNP, single nucleotide polymorphism; XBP1, x-box binding protein 1.

**Table 2:** GWAS on response to lithium in patients with BD.

Sample	Definition of Lithium Response	Main Findings	Ref.
Discovery: 458 patients with BD; replication: 359 patients with BD	Time to recurrence under lithium treatment	No genome-wide significant SNPs. Strongest association for a region on chromosome 10p15 (rs10795189, *p* = 5.5 × 10^−7^)	[58]
Discovery: 52 patients with BD; replication: 204 patients with BD	Alda scale	No genome-wide significant SNPs. Strongest signal for SNP rs11869731 (*p* = 7.21 × 10^−6^) located in the *ACCN1* gene	[59]
Discovery: 294 patients with BD I; replication: 100 and 24 patients with BD I	Alda scale	Two SNPs in LD (rs17026688, *p* =5.50 × 10^−37^ and rs17026651, *p* = 2.52 × 10^−37^), located in the intronic region of the *GADL1* gene, were significantly associated with lithium response	[56]
2698 and 1176 patients with BD with selfreported or clinically documented lithium response; 15583 HC	Self-reported and register data	No genome-wide significant SNPs when comparing R with NR. When comparing R with HC, significant association between rs116323614 (*p* = 2.74 × 10^− 8^) in the *SESTD1* gene and lithium responsive BD	[60]
Discovery: 2563 patients with BD; replication: 73 patients with BD	Alda scale	Four SNPs in LD on chromosome 21 (rs79663003, rs78015114, rs74795342, rs75222709) were associated with lithium response evaluated as a quantitative trait (lowest *p* for rs74795342, *p* = 3.3 × 10^−^^9^)	[55]

ACCN1, acid sensing ion channel subunit 2; BD I, bipolar disorder type 1; GADL1, glutamate decarboxylase like 1; HC, healthy controls; LD, linkage disequilibrium; NR, nonresponders R, responders; SESTD1, SEC14 and spectrin domain containing 1;SNP, single nucleotide polymorphism.

**Table 3:** Studies investigating epigenetic markers in cells derived from patients with BD treated with lithium or other human-derived cell lines exposed to lithium.

Epigenetic Marker	Cells	Method	Main Findings	Ref.
Epigenetic traits in cells derived from patients with BD treated with lithium				
Methylation of two *ARNTL* CpG island	Whole blood from 151 patients with BD (40 of which treated with lithium) and 66 HC	Pyrosequencing	Significant association between methylation of cg05733463 in the *ARNTL* gene and lithium (*p* = 0.005) or anticonvulsant (*p* = 0.002) intake	[85]
Global methylation	Leukocytes from 115 BD I on SGA therapy (73%) or mood stabilizer monotherapy (27%)	LUminometric Methylation Assay	Lower global methylation levels in BD patients treated with antipsychotics compared to those treated with mood stabilizers (*p* = 0.04)	[87]
Methylation of the *BNDF* promoter	PBMC from 94 patients with BD (24 of which treated with lithium) and 52 HC	Real time PCR	*BDNF* promoter hypomethylation in patients under therapy with lithium or valproate compared with other drugs (*p* < 0.05)	[82]
Methylation of the *BNDF* promoter	PBMC from 111 patients with BD type I, 50 with BD II and 43 with MDD	Real time PCR	Trend for *BDNF* promoter hypomethylation in patients under therapy with lithium or valproate compared with treatment with other drugs	[83]
Methylation of the *PDYN* promoter	PBMC from 54 patients with BD I, 45 with BD II and 42 HC	Real time PCR	Lower *PDYN* promoter methylation in patients treated with lithium and anticonvulsants compared with other drugs (*p* = 0.015)	[84]
Epigenetic traits in cells derived from patients characterized for lithium response				
Global methylation	Leukocytes from 29 patients with BD on lithium monotherapy, 11 on lithium + valproate, 22 on lithium + antipsychotics and 26 HC	ELISA	Decreased methylation in patients on lithium monotherapy compared to lithium + valproate or to controls (*p* = 0.036). No association between global methylation levels and lithium response	[88]
Genome-wide methylation	Whole blood from 172 patients with BD	Infinium Human Methylation27 and Human Methylation450K	Valproate was associated with higher values of the second PC of methylation levels (*p* = 0.02), while olanzapine and lithium with lower values (*p* = 0.05 for both). Overall, valproate and quetiapine but not lithium, were associated with altered methylation signatures after adjustment for drug related changes on cell type composition	[89]
Global methylation	LCLs from 14 patients with BD responders to lithium, 14 affected relatives, 16 unaffected relatives and 16 HC	ELISA	Lower methylation levels in patients with BD (*p* = 0.021) and their relatives compared with HC. In vitro treatment with lithium 0.75 mM for 1 week increased methylation levels in affected and unaffected relatives, but not in patients or HC	[90]
Genome-wide methylation	Peripheral blood cells from 15 patients with BD I responders to lithium and 11 non-responders	SeqCap Epi	111 DMRs between responders and non-responders (FDR < 0.05), significantly enriched in neuronal cell components. A total of 7 DMRs showed an AUC of 0.806 in the discrimination of responders and non-responders	[91]
Epigenetic changes induced by treatment with lithium in cell lines of human origin				
Genome-wide methylation	Human neuroblastoma SK-N-SH cells	Infinium Human Methylation27 BeadChip	Extensive methylation changes after in vitro lithium treatment (345 genes hypermethylated and 138 hypomethylated). Genes specifically altered by lithium were enriched for GO terms related to neuronal functions	[92]

ARNTL, aryl hydrocarbon receptor nuclear translocator like; AUC, area under the curve; BD, bipolar disorder; BD I, bipolar disorder type 1; BD II, bipolar disorder type 2; BDNF, brain derived neurotrophic factor; DMR, differentially methylated region; FDR, false discovery rate; GO, gene ontology; HC, healthy controls; LCL, lymphoblastoid cell lines; PBMC, peripheral blood mononuclear cells; PC, principal component; PDYN, prodynorphin; SGA, second generation antipsychotics.

**Table 4:** Studies investigating noncoding RNAs in cells or biofluids derived from patients with BD treated with lithium or other human-derived cell lines exposed to lithium.

Trait	Cells	Method	Main Findings	Ref.
miRNA levels in cells or biofluids derived from patients with BD treated with lithium				
13 miRNAs	LCLs from ten patients with BD and ten discordant sibilings treated with lithium 1 mM for 16 days	qRT-PCR	miR-221, miR-152, miR-155 and miR-34a were upregulated by lithium both at day 4 and 16 (*p* < 0.05)	[94]
miR-134	Plasma from 21 manic patients with BD treated with mood stabilizers (14 with lithium) and antipsychotics for 4 weeks	qRT-PCR	Plasma miR-134 levels were lower in patients compared with controls at baseline and significantly increased after 4 weeks of treatment (*p* < 0.001 for both)	[96]
let7-c	LCLs from 20 patients with BD treated with lithium 1–15 mM	qRT-PCR	Nonsignificant trend for higher expression of let7-c in LCLs from patients with BD NR compared to R to lithium (*p* = 0.1). No significant effect of in vitro lithium treatment	[97]
Genome-wide miRNA levels	LCLs from 16 patients with BD treated with lithium 1 mM for 1 week	Microarray, qRT-PCR	Several members of the let-7 family were downregulated by lithium in both R and NR	[98]
Genome-wide miRNA levels	LCLs from 20 patients with BD treated with lithium 1 mM for 1 week	NGS, qRT-PCR	miR-320a (FC = 0.55, FDR = 3.8 × 10^−8^) and miR-155-3p (FC = 2.27, FDR = 0.003) were down- and up-regulated, respectively, in R compared with NR	[99]
Genome-wide miRNA levels	LCLs from seven patients with BD who died by suicide, 11 at low risk of suicide and 12 controls treated with lithium 1 mM for 1 week	nCounter^®^ asssay, qRT-PCR	miR-4286 (FC = 1.16; FDR = 0.0018) and miR-186-5p (FC = 0.96, FDR = 0.0045) were increased and decreased, respectively, in LCLs from patients who died by suicide compared with patients at low risk and controls	[100]
miRNA levels in human-derived cells exposed to lithium				
miR-30a-5p	Human neuroblastoma cell line (SH-SY5Y) exposed to neurotoxic concentration of L-glutamate 2 mM, treated with lithium 1 mM or valproate 0.5 mM for 48 h	qRT-PCR	Increase in miR-30a-5p expression at 24 h in cells treated with L-glutamate (*p* < 0.01) or lithium, valproate and L-glutamate (*p* < 0.05). Decrease in miR-30-5p levels at 48 h after treatment with either L-glutamate (*p* < 0.01), or lithium + valproate (*p* < 0.001)	[101]
Genome-wide RNA	All-trans retinoic acid differentiated human neuroblastoma neuronal cell line (SK-N-SH) treated with lithium 1 mM for 1 week	NGS	Lithium was more likely to influence noncoding small nucleolar RNAs compared to miRNAs (*p* < 0.05)	[102]

BD, bipolar disorder; FC, fold change; FDR, false discovery rate; LCL, lymphoblastoid cell lines; NGS, next generation sequencing; NR, nonresponders; qRT-PCR, quantitative reverse transcription PCR; R, responders; Ref., reference.
